# Clinical outcomes of multivessel coronary artery disease patients revascularized by robot-assisted vs conventional standard coronary artery bypass graft surgeries in real-world practice

**DOI:** 10.1097/MD.0000000000023830

**Published:** 2021-01-22

**Authors:** Tzu-Hsiang Lin, Chi-Wei Wang, Ching-Hui Shen, Keng-Hao Chang, Chih-Hung Lai, Tsun-Jui Liu, Kuan-Ju Chen, Yu-Wei Chen, Wen-Lieng Lee, Chieh-Shou Su

**Affiliations:** a Cardiovascular Center, Taichung Veterans General Hospital; b Division of Cardiology, Asia University Hospital; c Department of Anesthesiology, Taichung Veterans General Hospital, Taichung; d Department of Medicine and Surgery, National Yang Ming University School of Medicine, Taipei; e Department of Internal Medicine, Cheng Ching Hospital, Taichung; f Institute of Clinical Medicine; g Department of Medicine, National Yang Ming University School of Medicine; h Department of Emergency Medicine, Taichung Veterans General Hospital, Taichung, Taiwan.

**Keywords:** coronary artery bypass graft surgery, multivessel coronary artery disease, robot-assisted coronary artery bypass graft surgery

## Abstract

The treatment of patients with multivessel coronary artery disease (MVD) by coronary stenting (PCI) and the “gold standard” conventional coronary-artery bypass grafting (C-CABG) has been well explored in the literature. However, the clinical outcomes of robot-assisted CABG (R-CABG) vs C-CABG in MVD patients in real-world practice were unknown. We aimed to study the clinical outcomes of MVD patients who underwent R-CABG (robotic MIDCAB) and C-CABG at our institution between January 2005 and December 2013.

A total of 516 MVD patients received CABG were recruited into this study. Among them, 281 patients received R-CABG and 235 patients underwent C-CABG. Patients in the R-CABG group were younger, and had fewer vessels with coronary artery disease (CAD), lower prevalence of chronic renal disease (CKD), higher left ventricular ejection fraction (LVEF), as well as lower Euro scores. The in-hospital and long-term mortalities were lower in the R-CABG group, but the incidences of target lesion revascularization (TLR), target vessel revascularization (TVR), myocardial infarction (MI), and stroke were not significantly different between the two groups. The long-term mortality was related to age, lower LVEF, and CKD, but not residual SYNTAX score, or completeness of revascularization. The revascularization modality (R-CABG vs C-CABG) was a borderline significantly independent predictor of long-term mortality (OR 1.76 [0.99–3.14], *P* = .055).

Our study concluded that R-CABG, in comparison with C-CABG, for MVD carried out in younger patients involved fewer clinical complexities was associated with lower in-hospital and long-term mortalities in real-world practice. However, the long-term rates of TLR, TVR, MI, and stroke were similar. The long-term mortality was correlated with age, lower LVEF, and CKD, where R-CABG remained a borderline significant predictor after correcting for confounding factors. R-CABG could be an effective alternative to C-CABG for MVD patients with fewer clinical complexities in real-world practice.

## 1 Introduction

Patients with multivessel coronary artery disease (MVD) have more comorbidities, greater likelihood of left ventricular dysfunction, and higher cardiovascular risks than those with single vessel disease. Both coronary artery bypass surgery (CABG) and percutaneous coronary intervention (PCI) have been found to be safe, and effective revascularization modalities.^[1–5]^ CABG with full arterialization for MVD has been the standard practice worldwide and achieves outstanding results.^[6,7]^ In recent years, robot-assisted surgeries have been increasingly applied to treat complex coronary artery disease (CAD),^[8–12]^ valvular heart disease (VHD),^[13,14]^ and congenital heart disease^[15,16]^ because they have the advantages of shorter wounds, shorter ICU and hospital stays, lower blood transfusion requirements, fewer post-operative complications, and better post-operative quality of life. In the literature, most robot-assisted CABG (R-CABG) was used for less complex CAD and CAD with lower morbidity.^[10,12,17]^ Up-to-date, the clinical outcomes of R-CABG in comparison with conventional CABG (C-CABG) for MVD in real-world practice have not been reported. Thus, we retrospectively retrieved, reviewed, and analyzed data from patients treated with R-CABG or C-CABG in our hospital.

## 2 Methods and materials

Consecutive patients with angiographically proven MVD who underwent R-CABG or C-CABG from January 2005 to December 2013 at our institute were retrospectively recruited into this study. Patients who presented with cardiogenic shock and arrest, had end-stage renal disease or history of CABG, or received other open heart surgeries involving valves, vessels, heart chamber, and congenital defects were excluded from this study. Patients receiving hybrid CABG with PCI were also excluded if some of the epicardial vessels were not revascularized by CABG. The choice of PCI or CABG was mainly determined by current guidelines, but was also at the discretion of attending physicians. As a general rule, patients with proven MVD with higher SYNTAX scores (≥33) were recommended to receive CABG as the first strategy, and for those with intermediate SYNTAX scores (≥22 and <33) either CABG or PCI was chosen based on a consensus among the patient, the family, and attending physicians. For patients who opted for surgery, the decision to perform C-CABG or R-CABG depended on clinical co-morbidities, willingness of the patient, and the patient's financial status. C-CABG and R-CABG were both carried out according to the standard practice at this institute. R-CABG (robotic MIDCAB at our institute) was performed using the Da Vinci robotic operation system under generalized anesthesia via three pencil-sized incisions along the left anterior axillary line over the 2nd, 4th, and 6th intercostal space. First, the cardiovascular surgeon harvested the left radial artery for free sequential grafts by the endoscopic method and the left internal mammary artery (LIMA) graft inside the chest, and performed pericardiotomy to expose the native coronary arteries. Then, an incision about 2.5 to 3 cm long was created over the 2nd intercostal space adjacent to the sternal bone and free radial graft was connected to the LIMA graft in an end-to-side (U- or Y-graft) fashion. The use of U- or Y-grafting depended on the length of LIMA and the number of sequential anastomosis to be made. In major cases, U-graft was adopted with the free end of LIMA hand sewn to the body of the free radial graft. An off-pump hand-sewn LIMA-LAD anastomosis was performed in an end-to-side manner and sequential LIMA-radial artery grafts were anastomosed to the diagonal artery, the left circumflex in a side-to-side manner and/or to the posterior descending artery in an end-to-side manner, depending on the territory of diseased coronary arteries, via an 8-cm left anterolateral thoracotomy which was in essence equivalent to robotic MIDCAB but was not totally endoscopic CABG (TE-CABG) and was performed on off-pump hearts.

C-CABG was performed using the standard approach with traditional sternotomy under general anesthesia. Briefly, the cardiovascular surgeon harvested the LIMA and performed pericardiotomy to expose the coronary arteries via the median sternotomy. Then, the surgeon harvested the left radial artery from the left forearm or the superficial femoral vein from the left femoral thigh, according to the surgeon's decision. Next, the abovementioned procedures for R-CABG were performed. The C-CABG procedure was either performed on the beating heart or on the arrested heart at the discretion of operators.

The medical records were reviewed in details, and the baseline demographic data, as well as in-hospital and long-term outcomes were retrieved, recorded, and analyzed. The study protocol was reviewed and approved by the Institutional Review Board/Ethics Committee of this institute.

### 2.1 Statistical analysis

Continuous variables are presented as mean ± SD, and frequencies and percentages of categorical variables. Differences in continuous variables were analyzed by Student's *t* test and Mann–Whitney *U* test. Categorical variables were analyzed by chi-square. Logistic regression analysis was used to determine the independent predictors for in-hospital and long-term mortalities, target lesion revascularization (TLR), target vessel revascularization (TVR), myocardial infarction (MI), stroke and lengths of intensive care unit (ICU)/total hospital stay. Variables with a *P*-value of <.10 in the univariate analysis were included in the multivariate analysis to rule out confounding factors. Between-group differences were considered statistically significant if *P* < .05. The SPSS 19.0 (SPSS Inc, Chicago, IL) software package was used for statistical analysis.

## 3 Results

### 3.1 Baseline characteristics of all patients with MVD

**Figure 1:**
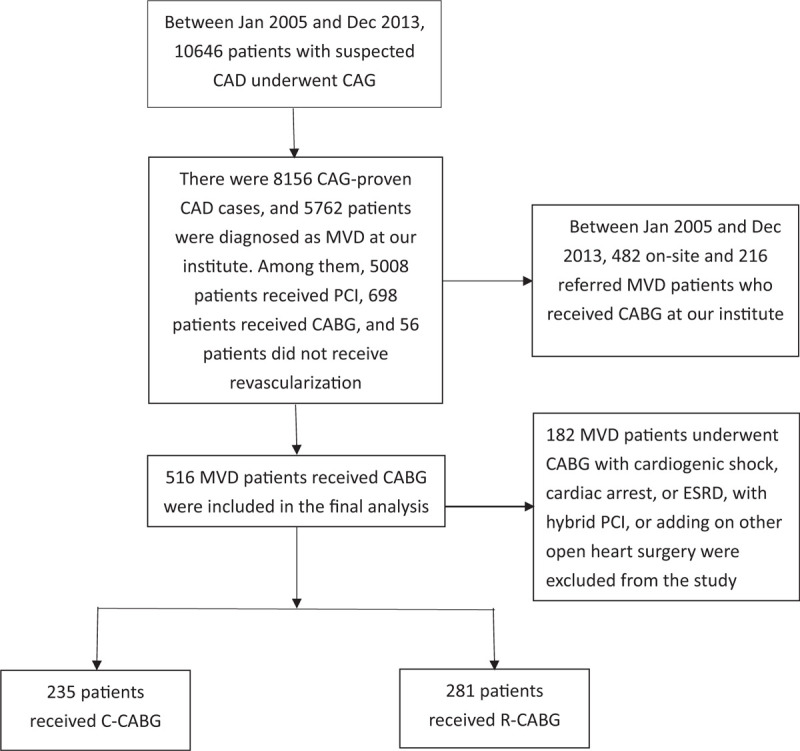
Flow chart of study design. A total of 516 patients with proven multiple-vessel coronary artery disease who received R-CABG, and C-CABG were included in the final analysis.

A flow chart of patients suspected CAD underwent coronary artery angiography and proven MVD disease with following interventions was shown as Figure 1. Among 5762 MVD disease patients, 516 patients were recruited into this study. Of these, 235 patients received C-CABG, and 281 patients received R-CABG. The baseline characteristics of these patients are shown in Table 1. Patients in the R-CABG group were younger, and had higher LVEF but had lower hemoglobin, lower Euro score and fewer diseased coronary arteries, despite having a greater prevalence of dyslipidemia under statin therapy. They also had lower prevalence rates of chronic kidney disease (CKD), and acute coronary syndrome (ACS) on admission compared to those of the C-CABG group.

**Table 1:** Demographic characteristics of coronary multiple vessel disease patients undergone robot-assisted vs contemporary standard coronary artery bypass grafting.

	R-CABG	C-CABG	
	n = 281	n = 235	*P*
Age, years	64.5 ± 11.2	66.8 ± 9.9	.042
Male, N (%)	229 (81.5)	184 (79.3)	.610
Diagnosis at admission			<.001
SCAD, N (%)	184 (65.5)	107 (46.1)	
ACS, N (%)	97 (34.5)	125 (53.9)	
CAD vessel numbers	2.7 ± 0.5	2.8 ± 0.4	.015
LM disease	80 (28.5)	73 (31.5)	.521
Hypertension, N (%)	232 (82.6)	186 (80.2)	.562
Diabetes mellitus, N (%)	134 (47.7)	126 (54.3)	.160
Smoking, N (%)	150 (53.4)	115 (49.6)	.441
Dyslipidemia, N (%)	180 (64.1)	105 (45.3)	<.001
CKD, N (%)	55 (19.6)	69 (29.7)	.010
PAD, N (%)	35 (12.5)	39 (16.8)	.204
Old MI, N (%)	41 (14.6)	39 (16.8)	.570
Prior PCI, N (%)	67 (23.8)	56 (24.1)	1.000
BMI, kg/m^2^	25.6 ± 3.7	25.5 ± 3.3	.853
Serum creatinine, mg/dL	1.4 ± 0.9	1.5 ± 1.0	.080
Hemoglobin, mg/dL	11.4 ± 1.8	12.4 ± 2.0	<.001
LVEF, %	51.9 ± 12.4	45.5 ± 13.6	<.001
SYNTAX score	31.4 ± 12.1	30.8 ± 11.9	.627
Euro score	5.7 ± 5.1	8.0 ± 4.9	<.001
Residual SYNTAX score	4.1 ± 4.4	3.9 ± 4.3	.644
Complete revascularization, N (%)	113 (40.2)	107 (46.1)	.224

### 3.2 In-hospital and post-hospital clinical outcomes

In- and post-hospital clinical outcomes are presented in Table 2. The need for intraaortic balloon pump (IABP) assistance was lower in the R-CABG group, and the lengths of ICU and hospital stays were also shorter. In addition, the in-hospital mortality was lower in the R-CABG group.

**Table 2:** In-hospital and post-hospital clinical outcomes of multiple vessel disease patients undergone robot-assisted vs contemporary standard coronary artery bypass grafting.

	R-CABG	C-CABG	
	N = 281	N = 235	*P*
In-hospital			
IABP assistance, N (%)	62 (22.0)	84 (36.2)	.001
ECMO assistance, N (%)	6 (2.1)	6 (2.6)	.966
ICU stay, day(s)	5.4 ± 10.8	11.4 ± 14.5	<.001
Hospital stay, day(s)	11.2 ± 12.7	17.4 ± 11.9	<.001
Death, N (%)	7 (2.5)	17 (7.3)	.018
Post-hospital events			
mean follow-up years	5.7 ± 3.0	5.0 ± 2.9	<.001
TLR, N (%)	25 (8.9)	15 (6.5)	.385
TVR, N (%)	25 (8.9)	15 (6.5)	.385
Any revascularization	31 (11.0)	19 (8.2)	.362
MI, N (%)	10 (3.6)	11 (4.7)	.633
Stroke, N (%)	12 (4.3)	13 (5.6)	.613
All-cause death, N (%)	23 (8.2)	48 (20.7)	<.001
Cardiac death, N (%)	8 (4.0)	15 (11.1)	.022

After hospital discharge, the R-CABG and C-CABG groups were followed up for a mean duration of 5.7 ± 3.0 and 5.0 ± 2.9 years, respectively (*P* < .001). The incidence rates of TLR, TVR, any revascularization, MI, stroke were not different between the two groups, but any-cause mortality and cardiac death in the C-CABG group were significantly higher than those in the R-CABG group.

### 3.3 Clinical predictors of in-hospital and long-term mortalities in all patients with MVD

Univariate and multivariate clinical predictors for in-hospital and long-term mortalities in all MVD patients who underwent CABG are presented in Tables 3 and 4. Multivariate logistic regression analysis showed that age, and need for extracorporeal membrane oxygenation (ECMO) assistance were independent predictors for in-hospital mortality, whereas age, lower LVEF, and CKD were independent predictors for long-term mortality. Total SYNTAX score, residual SYNTAX score, and completeness of revascularization were not predictors of long-term mortality, whereas revascularization modality (R-CABG vs C-CABG) was a borderline significant independent predictor of long-term mortality (odds ratio 1.76 [0.99–3.14], *P* = .055).

**Table 3:** Logistic regression analysis predictors for in-hospital mortality of all multivessel coronary artery disease patients.

	Univariate			Multivariate		
Variables	OR	95%CI	*P*	OR	95% CI	*P*
Age	1.07	1.02–1.13	.004	1.07	1.00–1.14	.036
Male	1.36	0.50–3.68	.546	—	—	—
Hypertension	1.90	0.44–8.12	.388	—	—	—
Diabetes mellitus	1.23	0.53–2.85	.628	—	—	—
Body mass index	0.91	0.79–1.04	.169	—	—	—
LVEF	0.96	0.93–0.99	.001	0.97	0.95–1.00	.059
Diagnosis of ACS	1.30	0.52–3.25	.569	—	—	—
LM disease	0.94	0.39–2.30	.899	—	—	—
CAD vessel numbers	1.21	0.45–3.27	.703	—	—	—
C-CABG vs R-CABG	1.40	0.54–3.63	.483	—	—	—
Dyslipidemia with statin	0.87	0.38–2.01	.750	—	—	—
Smoking	1.13	0.50–2.58	.764	—	—	—
Prior MI	0.38	0.09–1.61	.187	—	—	—
Prior PCI	1.54	0.63–3.76	.344	—	—	—
Hemoglobin	0.95	0.78–1.15	.597	—	—	—
Serum creatinine	1.21	0.96–1.53	.104	—	—	—
IABP assistance	4.18	1.63–10.72	.003	1.97	0.61–6.33	.256
ECMO assistance	22.29	9.12–54.47	<.001	12.40	4.17–36.89	<.001
Euro score	1.11	1.01–1.20	.022	0.96	0.86–1.08	.508
SYNTAX score	0.98	0.95–1.02	.328	—	—	—
Residual SYNTAX score	0.97	0.87–1.07	.519	—	—	—
Complete Revascularization	0.62	0.27–1.40	.246	—	—	—

**Table 4:** Logistic regression analysis predictors for long-term mortality of multivessel coronary artery disease patients.

	Univariate			Multivariate		
Variables	OR	95%CI	*P*	OR	95%CI	*P*
Age	1.09	1.06–1.12	<.001	1.07	1.03–1.11	.001
Male	0.71	0.40–1.23	.221	—	—	—
Hypertension	2.62	1.20–5.71	.016	1.91	0.81–4.52	.140
Diabetes mellitus	1.23	0.77–1.95	.392	—	—	—
Body mass index	0.86	0.80–0.93	<.001	0.97	0.89–1.05	.422
LVEF	0.94	0.93–0.96	<.001	0.96	0.94–0.98	<.001
Hemoglobin	0.85	0.75–0.97	.012	0.97	0.84–1.12	.683
CKD	1.71	1.49–1.97	<.001	1.41	1.18–1.69	<.001
CAD vessel number	1.76	0.98–3.16	.058	—	—	—
Diagnosis of ACS	3.37	2.07–5.49	<.001	1.39	0.49–3.95	.536
LM disease	0.62	0.35–1.12	.114	—	—	—
C-CABG vs R-CABG	3.27	1.98–5.39	<.001	1.76	0.99–3.14	.055
Dyslipidemia on statin	0.59	0.37–0.94	.026	0.85	0.50–1.44	.539
Smoker	0.79	0.49–1.25	.312	—	—	—
Prior MI	1.49	0.84–2.63	.173	—	—	—
Prior PCI	1.63	0.99–2.69	.053	—	—	—
Euro score	1.21	1.15–1.26	<.001	1.02	0.90–1.16	.737
SYNTAX score	1.01	0.99–1.03	.434	—	—	—
Residual SYNTAX score	1.03	0.97–1.09	.388	—	—	—
Complete Revascularization	0.98	0.58–1.66	.942	—	—	—

### 3.4 Clinical predictors of ICU/total hospital stays in all patients with MVD

The multivariate linear regression analysis model showed that the only independent determinant for durations of ICU and total hospital stays was the revascularization modality, that is, age, body mass index, LVEF, ACS, CKD, and Euro score showed no correlation with ICU or hospital stays (Tables 5 and 6).

**Table 5:** Multivariate linear regression analysis predictors for ICU stay of all multivessel coronary artery disease patients.

	Coefficients				
Independent variables	*B*	SE	*t*	*P*	95% CI
Constant	−1.229	3.25	−0.378	.706	(−7.63, 5.16)
Age	0.043	0.04	1.201	.230	(−0.03, 0.11)
Body mass index	0.041	0.08	0.521	.603	(−0.11, 0.20)
LVEF	−0.038	0.25	−1.564	.119	(−0.09, 0.01)
Diagnosis of ACS	0.944	1.04	0.904	.366	(−1.11, 2.99)
Revascularization modality	2.003	0.56	3.549	<.001	(0.89, 3.11)
(C-CABG vs R-CABG)					
Dyslipidemia	−1.020	0.558	−1.827	.068	(−2.12, 0.08)
CKD	−0.370	0.67	−0.550	.582	(−1.69, 0.95)
Euro score	0.195	0.13	1.478	.140	(−0.06, 0.45)

**Table 6:** Multivariate linear regression analysis predictors for total hospital stay of all multivessel coronary artery disease patients.

	Coefficients				
Independent variables	*B*	SE	*t*	*P*	95% CI
Constant	9.928	6.34	1.567	.118	(−2.52, 22.38)
Age	0.010	0.07	0.144	.886	(−0.12, 0.14)
Sex	−1.453	1.32	−1.11	.270	(−4.04, 1.13)
Body mass index	−0.173	0.15	−1.16	.248	(−0.46, 0.12)
LVEF	−0.047	0.05	−1.03	.306	(−0.14, 0.04)
Diagnosis of ACS	−1.382	2.02	−0.68	.494	(−5.35, 2.59)
Revascularization modality	4.779	1.05	4.56	<.001	(2.72, 6.84)
(C-CABG vs R-CABG)					
Prior MI	2.417	1.41	1.71	.087	(−0.35, 5.19)
CKD	0.497	1.28	0.39	.697	(−2.01, 3.01)
Euro score	0.643	0.26	2.50	.013	(0.14, 1.15)

### 3.5 Clinical predictors of major adverse cardiac and cardiovascular events

The univariate and multivariate clinical predictors for major adverse cardiac and cardiovascular events (MACCEs), including TLR, TVR, MI, and stroke are presented in Tables 7–10. DM was the only independent factor that predicted TLR and TVR. Age, DM, and lower LVEF were independent predictors of MI, whereas there were no independent predictors of stroke during the follow-up.

**Table 7:** Logistic regression analysis predictors for target lesion revascularization of all multivessel coronary artery disease patients.

	Univariate			Multivariate		
Variables	OR	95%CI	*P*	OR	95% CI	*P*
Age	1.03	1.00–1.07	.050	1.02	0.98–1.07	.267
Male	0.47	0.23–0.94	.032	0.74	0.33–1.67	.470
Hypertension	1.19	0.56–2.50	.650	—	—	—
Diabetes mellitus	2.44	1.26–4.74	.008	2.42	1.19–4.93	.015
Body mass index	0.97	0.89–1.07	.561	—	—	—
LVEF	0.99	0.97–1.01	.332	—	—	—
Diagnosis of ACS	1.22	0.64–2.34	.547	—	—	—
LM disease	1.88	1.00–3.55	.050	1.70	0.86–3.36	.125
CAD vessel numbers	1.28	0.65–2.52	.482	—	—	—
C-CABG vs R-CABG	1.05	0.54–2.04	.885	—	—	—
Dyslipidemia with statin	1.46	0.75–2.85	.269	—	—	—
Smoking	0.74	0.40–1.38	.349	—	—	—
Prior MI	1.25	0.52–2.99	.612	—	—	—
Prior PCI	1.76	0.89–3.49	.103	—	—	—
Hemoglobin	0.75	0.64–0.89	.001	0.85	0.69–1.04	.107
Serum creatinine	1.57	1.08–2.28	.017	1.33	0.86–2.06	.195
Euro score	1.07	1.00–1.14	.041	1.01	0.93–1.09	.865
SYNTAX score	1.01	0.98–1.03	.440	—	—	—
Residual SYNTAX score	1.03	0.96–1.08	.432	—	—	—
Complete revascularization	1.15	0.59–2.22	.683	—	—	—

**Table 8:** Logistic regression analysis predictors for target vessel revascularization of all multivessel coronary artery disease patients.

	Univariate			Multivariate		
Variables	OR	95%CI	*P*	OR	95% CI	*P*
Age	1.03	1.00–1.07	.050	1.02	0.98–1.07	.267
Male	0.47	0.23–0.94	.032	0.74	0.33–1.67	.470
Hypertension	1.19	0.56–2.50	.650	—	—	—
Diabetes mellitus	2.44	1.26–4.74	.008	2.42	1.19–4.93	.015
Body mass index	0.97	0.89–1.07	.561	—	—	—
LVEF	0.99	0.97–1.01	.332	—	—	—
Diagnosis of ACS	1.22	0.64–2.34	.547	—	—	—
LM disease	1.88	1.00–3.55	.050	1.70	0.86–3.36	.125
CAD vessel numbers	1.28	0.65–2.52	.482	—	—	—
C-CABG vs R-CABG	1.05	0.54–2.04	.885	—	—	—
Dyslipidemia on statin	1.46	0.75–2.85	.269	—	—	—
Smoking	0.74	0.40–1.38	.349	—	—	—
Prior MI	1.25	0.52–2.99	.612	—	—	—
Prior PCI	1.76	0.89–3.49	.103	—	—	—
Hemoglobin	0.75	0.64–0.89	.001	0.85	0.69–1.04	.107
Serum creatinine	1.57	1.08–2.28	.017	1.33	0.86–2.06	.195
Euro score	1.07	1.00–1.14	.041	1.01	0.93–1.09	.865
SYNTAX score	1.01	0.98–1.04	.440	—	—	—
Residual SYNTAX score	1.03	0.96–1.11	.432	—	—	—
Complete Revascularization	1.15	0.59–2.22	.683	—	—	—

**Table 9:** Logistic regression analysis predictors for myocardial infarction of all multivessel coronary artery disease patients.

	Univariate			Multivariate		
Variables	OR	95%CI	*P*	OR	95% CI	*P*
Age	1.07	1.02–1.12	.004	1.09	1.02–1.17	.010
Male	0.31	0.13–0.76	.010	0.48	0.17–1.32	.153
Hypertension	1.34	0.45–3.98	.602	—	—	—
Diabetes mellitus	6.47	1.90–21.99	.003	6.52	1.77–24.03	.005
Body mass index	0.87	0.76–0.99	.038	1.01	0.87–1.16	.916
LVEF	0.96	0.93–0.99	.005	0.95	0.91–0.99	.013
Diagnosis of ACS	1.82	0.77–4.31	.171	—	—	—
LM disease	1.03	0.40–2.67	.948	—	—	—
CAD vessel numbers	2.97	0.87–10.15	.082	—	—	—
C-CABG vs R-CABG	1.92	0.80–4.58	.142	—	—	—
Dyslipidemia	1.12	0.46–2.70	.807	—	—	—
Smoking	0.48	0.20–1.15	.100	—	—	—
Prior MI	1.07	0.31–3.65	.913	—	—	—
Prior PCI	1.14	0.42–3.10	.804	——	—	—
Hemoglobin	0.70	0.55–0.89	.003	0.79	0.60–1.03	.086
Serum creatinine	1.39	0.83–2.30	.208	—	—	—
Euro score	1.14	1.05–1.24	.002	0.97	0.86–1.09	.588
SYNTAX score	1.02	0.98–1.05	.256	—	—	—
Residual SYNTAX score	1.03	0.94–1.13	.514	—	—	—
Complete revascularization	1.01	0.42–2.41	.991	—	—	—

**Table 10:** Logistic regression analysis predictors for stroke of all multivessel coronary artery disease patients.

	Univariate			Multivariate		
Variables	OR	95%CI	*P*	OR	95% CI	*P*
Age	1.04	1.00–1.08	.046	1.02	0.97–1.08	.377
Male	1.02	0.35–2.97	.975	—	—	—
Hypertension	2.33	0.70–7.80	.169	—	—	—
Diabetes mellitus	1.19	0.54–2.62	.663	—	—	—
Body mass index	1.10	1.00–1.22	.061	—	—	—
LVEF	0.97	0.95–1.00	.076	—	—	—
Diagnosis of ACS	2.35	1.06–5.22	.036	1.29	0.31–6.01	.687
LM disease	0.99	0.41–2.38	.984	—	—	—
CAD vessel numbers	1.35	0.56–3.26	.506	—	—	—
C-CABG vs R-CABG	1.64	0.74–3.65	.224	—	—	—
Dyslipidemia	1.27	0.56–2.89	.566	—	—	—
Smoking	0.84	0.38–1.84	.659	—	—	—
Prior MI	1.26	0.43–3.68	.675	—	—	—
Prior PCI	1.19	0.47–2.99	.709	—	—	—
Hemoglobin	1.00	0.81–1.23	.986	—	—	—
CKD	1.59	0.59–4.27	.356	—	—	—
Euro score	1.12	1.03–1.20	.006	1.06	0.90–1.26	.474
SYNTAX score	0.992	0.95–1.03	.659	—	—	—
Residual SYNTAX score	0.99	0.90–1.09	.885	—	—	—
Complete revascularization	0.73	0.32–1.64	.445	—	—	—

## 4 Discussion

The main findings of our study comparing the clinical outcomes of R-CABG vs C-CABG for MVD patients are as follows:

1. R-CABG group patients were younger, had fewer clinical/angiographic complexities, had shorter lengths of ICU/hospital stays, and had lower mortality rates during hospitalization and a mid-term follow-up period than C-CABG patients;
2. age and need for ECMO assistance were independent predictors of in-hospital mortality;
3. ICU/total hospital stays were related to revascularization modality only;
4. DM was the only independent factor of TLR and TVR, whereas age, DM, and lower LVEF were independent predictors of MI;
5. Age, lower LVEF, and CKD were independent predictors of long-term mortality, but SYNTAX score, residual SYNTAX score, and completeness of revascularization were not predictors of long-term mortalities, TLR, TVR, MI, or stroke.

The revascularization modality (R-CABG vs C-CABG) remained a borderline significantly independent predictor of mortality in the follow-up.

Both CABG and PCI are effective treatments for patients with MVD, but the treatment of choice for MVD remains a subject of debate.^[1–5,18]^ CABG has proven to be superior to PCI in terms of the need for repeat interventions^[1,3,4,18]^ and long-term survivals, especially in complex MVD patients,^[6]^ although the choice of therapy in real-world practice is affected by many other factors. With advances in surgical devices and techniques, open-wound surgeries have gradually been replaced by endoscopic surgeries, such as robot-assisted surgeries. Over the past two decades, robot-assisted cardiovascular surgeries using the Da Vinci system, which combines the advantages of two revascularization modalities to provide smaller wounds, less rib retraction, less post-operative pain, faster return to normal activities and a positive impact on quality of life,^[8,19]^ has been increasingly used worldwide to treat congenital heart disease,^[15,16]^ VHD,^[13,14]^ and CAD.^[8–12]^ In technical terms, R-CABG could be classified as totally endoscopic (TE-R-CABG) or robot-assisted minimally-invasive (robotic MIDCAB), depending on the way the grafts are anastomosed to the native arteries. Due to concerns about anastomosis patency in the early stage, only robotic MIDCAB is employed in our institute. The results of R-CABG have been quite encouraging with a number of persuasive advantages that include lower hospital cost,^[20–22]^ less administration of analgesics in the post-operative course,^[23]^ shorter ICU/hospital stays,^[22]^ and fewer peri-/post-operative MACCEs.^[21,22,24]^ R-CABG is typically used for treating simple rather than complex CAD, in the majority of cases, because it is more time-consuming and technically demanding than C-CABG when it comes to performing multiple and distal anastomoses. However, the effectiveness and safety of R-CABG in comparison with C-CABG in complex MVD patients, especially in real-world practice, remain unclear. Our results corroborated this general trend as R-CABG patients were younger and had fewer clinical comorbidities, as well as lower severity of CAD severity and ACS than C-CABG patients. Therefore, differences in risk factors at study entry might have confounded the clinical outcomes of R-CABG vs C-CABG. In the present study, the less invasive surgical modality, R-CABG, was associated with shorter ICU and hospital stays. After adjusting for confounding factors, such as age, gender, clinical comorbidities, heart function and diagnosis of ACS, R-CABG remained a significant predictive factor of shorter hospital stays. In the literature, the average hospital stays for C-CABG was 6 to 9 days.^[6,21,24]^ The reason that the duration of stay was longer at our hospital might be explained by the fact that our study population was exclusively composed of patients with true MVD and ACS accounted for over half of the patients. Furthermore, hospital costs tend to be lower in Taiwan compared to costs in Western countries, so pressure on hospital beds may be lower, which may have resulted in longer length of hospital stay in our patients. Our in-hospital mortality rate was somewhat higher than that reported in the literature. This might be ascribed to the inclusion of ACS patients, who comprised more than half of the study population, and their Euroscores were much higher.

In the present study, R-CABG was associated with lower in-hospital mortality, but not an independent risk predictor. Despite the lower in-hospital MACE and mortality associated with R-CABG reported in the literature, even in the setting of MVD and multiple grafting, all of the procedures were conducted well-selected patients and none of the studies employed randomized design.^[24]^ In the few studies showing better short-term results of R-CABG over C-CABG, the sample size was small and low in clinical/anatomic complexities.^[12]^ In the present study, only robotic MIDCAB was used. Whether the outcomes of robotic MIDCAB differ from those of TE-R-CABG remains unknown. Nonetheless, our study results confirm that robotic MIDCAB offers advantages over the conventional approach in real-world practice. Interestingly, R-CABG was found to be a borderline significant predictor of long-term mortality in our study. Whether R-CABG could be an independent predictor of lower long-term mortality remains to be determined in larger investigations or randomized studies.

The treatment goal of MVD revascularization is to reduce angina, and ischemic heart failure and to improve patient survival. Once the safety and efficacy of PCI or CABG for treatment of patients with MVD had been established, the completeness of revascularization of MVD became another matter of concern, and remains a subject of debate.^[25–33]^ In the present study, completeness of revascularization was not a predictor of in-hospital mortality, total follow-up mortality, MI, TLR, TVR, or stroke. However, the residual SYNTAX scores were only 4.1 ± 4.4 and 3.9 ± 4.3 in the R-CABG and C-CABG groups, respectively, and were relatively low after either revascularization. Given that complete revascularization was only achieved in 40.2% and 46.1% in R-CABG and C-CABG groups, respectively, this result might imply that residual lesions and ischemia in patients were insufficient to notably benefit from revascularization of major epicardial coronary arteries, especially the LADs. Future larger, randomized and prospective studies are needed to clarify this issue.

In conclusion, R-CABG (robotic MIDCAB) for complex MVD in real-world practice was used to treat patients with fewer comorbidities, as well as lower severity of CAD and surgical risks, and had lower in-hospital and long-term mortalities, and similar MACCEs, in comparison with patients who underwent C-CABG. In the multivariate analysis, residual SYNTAX score, completeness of revascularization and revascularization modality were not independent predictors of in-hospital mortality, total mortality, TLR, TVR, MI, or stroke during follow-up in real-world practice. R-CABG appeared to be an effective and efficient alternative to C-CABG for the treatment of patients with complex MVD with backgrounds of fewer comorbidities, and lower CAD severity and surgical risks.

### 4.1 Study limitations

There were some limitations in this study. First, this was an observational, retrospective, and non-randomized study, and therefore subject to all the limitations inherent in the study design. Second, the choice of C-CABG or R-CABG was primarily based on co-morbidities, the patient's and the family's preference as well as their financial capability, rather than random assignment. Thus, there were selection biases and significant differences in background co-morbidities between the groups at study entry. As a consequence, multivariate logistic regression analysis was performed to adjust for confounding factors. R-CABG demands special surgical skills and certain patient pre-requirements, whereas C-CABG is the standard procedure for MVD, and therefore we think the investigated population in the present study and the design do indeed reflect real-world practice, as such, our conclusions are pertinent to typical clinical settings. However, the two cohorts in this study were relatively small. Larger randomized trials are needed to confirm our findings and to better establish the impact of R-CABG on the long-term outcomes of patients with complex MVD compared with those achieved by conventional CABG.

## Author contributions

**Conceptualization:** Chih-Hung Lai, Wen-Lieng Lee.

**Data curation:** Keng-Hao Chang, Chih-Hung Lai, Kuan-Ju Chen.

**Formal analysis:** Ching-Hui Shen.

**Investigation:** Tzu-Hsiang Lin, Chi-Wei Wang, Ching-Hui Shen, Keng-Hao Chang, Tsun-Jui Liu.

**Methodology:** Wen-Lieng Lee, Chieh-Shou Su.

**Resources:** Ching-Hui Shen, Kuan-Ju Chen, Yu-Wei Chen.

**Supervision:** Tsun-Jui Liu.

**Visualization:** Tzu-Hsiang Lin, Chih-Hung Lai, Tsun-Jui Liu, Yu-Wei Chen.

**Writing – original draft:** Tzu-Hsiang Lin, Chi-Wei Wang, Chieh-Shou Su.

**Writing – review & editing:** Wen-Lieng Lee, Chieh-Shou Su.
